# Parliament and the Profession

**Published:** 1916-05-27

**Authors:** 


### PARLIAMENT AND THE PROFESSION.

### Medical Officers and Military Rank.

In view of the fact that it is now proposed to give commissions and grant military rank to officers of Volunteer corps, Captain Douglas Hall asked whether the War Office was prepared to grant similar privileges to medical officers in charge of voluntary military hospitals. Mr. Tennant's reply was that commissions had already been given to all medical men doing work which rendered military rank necessary. It was not proposed to recommend any further extension of the granting of temporary commissions in the Royal Army Medical Corps except under this principle.

### War Prisoners in Swiss Sanatoria.

At last the arrangements for the transfer of selected British prisoners from Germany to Switzerland are reaching their final stage. Replying to Mr. Theodore Taylor, Sir Edward Grey stated that arrangements were being made to intern the British prisoners at Château d'Oex. Four Swiss Medical Commissions, consisting of two medical officers each, started for this country on May 20 to examine the German prisoners, and the Swiss Medical Commission which is to examine the British prisoners in Germany left Berne on the same date.

### Local Authorities and the Treatment of Venereal Diseases.

The President of the Local Government Board announced that he proposed to issue immediately a circular letter to local authorities explaining the arrangements which are required for the provision of facilities for the diagnosis and treatment of these diseases, and the conditions on which grants will be made for this purpose.

### Other Matters of Interest.

Makers of Artificial Limbs and Military Service.—Should makers of artificial limbs be exempt from military service? That, in effect, was the question asked by Mr. Jowett, based upon an individual case in which a person engaged in this occupation had been refused exemption by a local Tribunal. The reply was that the Government could not interfere with the decision of a local Tribunal in a case of this kind. In view of the fact that the business of making artificial limbs has now become one of great national concern, it is important that those who are skilled in the business should receive special consideration when their applications for exemption from military service are under review.

Medical Boards.—Replying to Sir Ryland Adkins, Mr. Tennant stated that the Medical Boards by which men previously rejected for the Army on medical grounds will be examined are those which have been appointed and are working in each recruiting area. The presidents are appointed by the War Office and the members are appointed in the commands.

Supply of Milk to Haslar Hospital.—The circumstances which led to the cancellation of the contract with the firm which had been supplying milk to Haslar Hospital was the subject of a statement by Dr. Macnamara in reply to a question asked by Mr. W. Nicholson. On three occasions during the last three years this firm has been discovered by the Hampshire County Council in the act of delivering impoverished milk, and it was to be inferred from Mr. Nicholson's question that the officer in charge of the hospital seemed to resent outside interference. Dr. Macnamara explained, however, that the hospital authorities had very efficient arrangements for supervising the milk supply, but the Admiralty welcomed any assistance which the County Council inspectors were able to give in the ordinary course of their duties. The arrangements suggested by the officer in charge of the hospital were intended solely to avoid any overlapping between the two authorities.

